# RAD54L2 counters TOP2-DNA adducts to promote genome stability

**DOI:** 10.1126/sciadv.adl2108

**Published:** 2023-12-06

**Authors:** Giuseppina D’Alessandro, David A. Morales-Juarez, Sean L. Richards, Karin C. Nitiss, Almudena Serrano-Benitez, Juanjuan Wang, John C. Thomas, Vipul Gupta, Andrea Voigt, Rimma Belotserkovskaya, Chen Gang Goh, Anne Ramsay Bowden, Yaron Galanty, Petra Beli, John L. Nitiss, Guido Zagnoli-Vieira, Stephen P. Jackson

**Affiliations:** ^1^ Cancer Research UK Cambridge Institute, University of Cambridge, Cambridge, UK.; ^2^ The Gurdon Institute and Department of Biochemistry, University of Cambridge, Cambridge, UK.; ^3^ UIC College of Pharmacy, Rockford, IL, USA.; ^4^ Institute of Molecular Biology (IMB), Chromatin Biology & Proteomics, Mainz, Germany.; ^5^ Institute of Developmental Biology and Neurobiology (IDN), Johannes Gutenberg-Universität, Mainz, Germany.

## Abstract

The catalytic cycle of topoisomerase 2 (TOP2) enzymes proceeds via a transient DNA double-strand break (DSB) intermediate termed the TOP2 cleavage complex (TOP2cc), in which the TOP2 protein is covalently bound to DNA. Anticancer agents such as etoposide operate by stabilizing TOP2ccs, ultimately generating genotoxic TOP2-DNA protein cross-links that require processing and repair. Here, we identify RAD54 like 2 (RAD54L2) as a factor promoting TOP2cc resolution. We demonstrate that RAD54L2 acts through a novel mechanism together with zinc finger protein associated with tyrosyl-DNA phosphodiesterase 2 (TDP2) and TOP2 (ZATT/ZNF451) and independent of TDP2. Our work suggests a model wherein RAD54L2 recognizes sumoylated TOP2 and, using its ATPase activity, promotes TOP2cc resolution and prevents DSB exposure. These findings suggest RAD54L2-mediated TOP2cc resolution as a potential mechanism for cancer therapy resistance and highlight RAD54L2 as an attractive candidate for drug discovery.

## Teaser

RAD54L2 resolves TOP2-DNA adducts and protects cells from anti-cancer drugs such as etoposide.

## INTRODUCTION

DNA topoisomerases moderate DNA topology during replication, transcription, recombination, and chromatin remodeling (*1*). Topoisomerase 2α (TOP2α) and TOP2β are unique among eukaryotic topoisomerases in their ability to form a transient DNA double-strand break (DSB) intermediate termed the TOP2 cleavage complex (TOP2cc), in which TOP2 is covalently bound to the DSB through a 5′-phosphotyrosyl bond (*2*). Following DNA unwinding or untangling, a religation reaction occurs, producing a relaxed DNA product (*3*). Widely used chemotherapeutic drugs, such as etoposide, inhibit DNA religation and form abortive TOP2ccs (*4*–*6*). TOP2ccs are primarily processed by tyrosyl-DNA phosphodiesterase 2 (TDP2), which hydrolyzes the 5′-phosphotyrosyl bond between TOP2 and DNA (*7*). This linkage is accessible after proteolytic degradation of the TOP2cc by the ubiquitin-proteasome system or specialized proteases (*8*–*10*). Alternatively, attachment of small ubiquitin-like modifier (SUMO) proteins to abortive TOP2ccs by zinc finger protein associated with TDP2 and TOP2 (ZATT/ZNF451) enables direct recruitment of TDP2 to the intact TOP2cc through TDP2’s split SUMO-interacting motifs (SIMs) to mediate TOP2 removal (*11*). Ultimately, processing of abortive TOP2ccs converges in the release of a DSB that can be religated through canonical nonhomologous end-joining (NHEJ) (*12*). Distinct from TDP2-mediated repair, abortive TOP2ccs can also be directly processed by specialized nucleases that “trim” DNA ends and lead to DSB repair by NHEJ or homologous recombination (HR) (*13*, *14*). The complexity of repairing TOP2-mediated DNA damage raises the prospect of as-yet uncharacterized proteins being involved in these events.

## RESULTS

### Focused CRISPR-Cas9 screens identify RAD54L2 as a factor whose loss confers hypersensitivity to etoposide

**Fig. 1.**
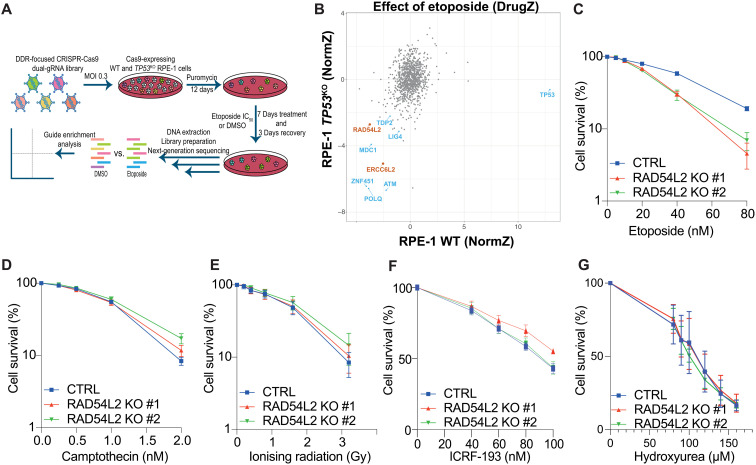
Focused CRISPR-Cas9 screens identify factors whose loss imparts etoposide hypersensitivity. (**A**) Layout of CRISPR-Cas9 screens in WT and *TP53*^KO^ RPE-1 cells [multiplicity of infection (MOI), drug concentration reducing cell viability by 50% (IC_50_)]. Figure generated with BioRender. (**B**) Biplot showing gene enrichment scores (NormZ) upon etoposide treatment in WT (*x* axis) and *TP53*^KO^ (*y* axis) RPE-1 cells. Blue or orange circles indicate known or previously unknown factors, respectively, to affect etoposide sensitivity. (**C** to **G**) MTT cell viability assays of two HAP1 *RAD54L2*^KO^ clones upon treatment with etoposide (C), camptothecin (D), ionizing radiation (E), ICRF-193 (F), or hydroxyurea (G). *n* = 3 independent experiments. Bars represent means ± SEM.

To identify factors involved in TOP2 biology, we performed CRISPR-Cas9 gene inactivation screens in wild-type (WT) and tumor protein 53 (*TP53*) knockout (*TP53*^KO^) human retinal pigment epithelial (RPE-1) cells using a focused dual-guide RNA library targeting 852 DNA damage response (DDR)–related genes (Fig. 1A) (*15*). In addition to identifying factors whose loss is known to cause etoposide hypersensitivity, such as TDP2, ZNF451, and ataxia telangiectasia mutated (ATM), we identified interesting candidates in both WT and *TP53*^KO^ RPE-1 cell screens (Fig. 1B). These included RAD54 like 2 (RAD54L2) and ERCC excision repair 6 like 2 (ERCC6L2), two poorly characterized putative DNA helicases.

To validate these results, we tested the sensitivity of *ERCC6L2*^KO^ or *RAD54L2*^KO^ human HAP1 cells (fig. S1A) to etoposide and various other DNA damaging agents. In line with recent reports suggesting a role for ERCC6L2 in the DDR (*16*–*19*), HAP1 *ERCC6L2*^KO^ cells were hypersensitive to etoposide (fig. S1B), as well as to other DNA damaging agents, including camptothecin and ionizing radiation (fig. S1, C and D). In stark contrast, *RAD54L2*^KO^ clones were hypersensitive to etoposide compared to RAD54L2-proficient parental control (CTRL) (Fig. 1C) but were not hypersensitive to the topoisomerase I poison camptothecin (Fig. 1D), ionizing radiation (Fig. 1E), or any of the other chemotherapeutic agents that we tested (fig. S1, E to H). Given that *RAD54L2*^KO^ cells were specifically sensitive to etoposide, we decided to focus on this factor for the rest of the study.

We next performed viability assays to test the impact of RAD54L2 on cell viability in the presence of the TOP2 catalytic inhibitor ICRF-193 (*6*). We observed that, as reported for *ZNF451^KO^* cells (*16*, *20*), *RAD54L2*^KO^ cells were not more sensitive to ICRF-193 than CTRL cells, suggesting that, like ZNF451, RAD54L2 does not play a prominent role in resolving lesions caused by this compound (Fig. 1F). We also observed that *RAD54L2*^KO^ clones were not hypersensitive to hydroxyurea (Fig. 1G), thus excluding a potential role in promoting replication fork reversal, as recently proposed for ZNF451 (*21*). To extend our findings, we generated *RAD54L2*^KO^ clones in RPE-1 cells (fig. S1I) and confirmed their hypersensitivity toward etoposide treatment (fig. S1J). To validate our findings in a third cell line and with a different experimental approach, we performed competitive cell growth assays in human osteosarcoma (U2OS) cells stably expressing Cas9. Briefly, we transduced the cells with two different single guide RNAs (sgRNAs) targeting *RAD54L2* and monitored the relative growth of the edited populations over time in the presence of selected doses of etoposide. We observed that sgRNA-mediated depletion of RAD54L2 hypersensitized U2OS cells to etoposide treatment (fig. S1, K and L).

### RAD54L2 binds SUMO and TOP2α/β and requires its SIMs to interact with SUMO-modified TOP2α/β

**Fig. 2.**
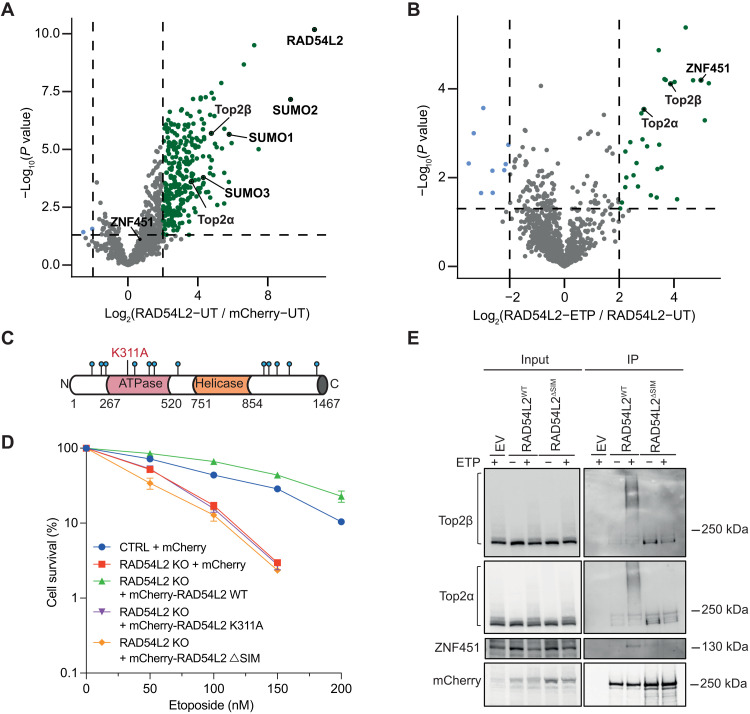
RAD54L2 binds SUMO and TOP2α/β and requires its SIMs to interact with SUMO-modified TOP2α/β. (**A** and **B**) Volcano plots showing RAD54L2 interactors identified by IP-MS in untreated (UT) cells (A) or etoposide (ETP)–treated cells. (B) Green dots bordered by dotted lines indicating *P* values ≤0.05 and log_2_ fold changes ≥2 are significant interactors. (**C**) Schematic of the putative domains and relevant properties of RAD54L2. The K311A catalytic site mutation is labeled, and the SIMs are highlighted in blue dots. (**D**) Clonogenic survival assays of control (CTRL) cells or *RAD54L2*^KO^ cells complemented with vectors expressing mCherry, mCherry-RAD54L2^WT^, mCherry-RAD54L2^K311A^ mutant, or mCherry-RAD54L2^ΔSIM^ mutant; *n* = 3 independent experiments. Bars represent means ± SEM. (**E**) Coimmunoprecipitation (IP) of mCherry from extracts of untreated or etoposide treated RPE-1 cells stably expressing mCherry (EV), mCherry-RAD54L2^WT^, or mCherry-RAD54L2^ΔSIM^ mutant. This experiment was repeated six times with similar results.

To further explore the link between RAD54L2 and TOP2 biology and identify RAD54L2 interactors, we stably expressed mCherry-RAD54L2 in RPE-1 cells grown in the presence or absence of etoposide and then performed immunoprecipitation experiments followed by mass spectrometry (IP-MS; fig. S2A). We observed that RAD54L2 interacts with TOP2α, TOP2β, SUMO1, SUMO2, and SUMO3 in untreated conditions (Fig. 2A). Pathway enrichment analysis of enriched proteins using PANTHER GOslim biological process annotations indicated the most enriched biological process as “DNA unwinding involved in DNA replication” (table S4), a process that introduces topological stress and is often resolved by TOP2 enzymes (*22*). Moreover, there was a substantial enrichment of interactors in the DNA replication pathway (fig. S2B). The interactions of RAD54L2 with TOP2α and TOP2β became further accentuated upon etoposide treatment (Fig. 2B). In addition, pronounced interaction with the SUMO E3 ligase ZNF451 was observed only upon etoposide treatment. Collectively, the IP-MS experiments suggested an interplay between sumoylation and the ability of RAD54L2 to bind TOP2α, TOP2β, and ZNF451. In line with our data, recent studies have identified RAD54L2 as a strong SUMO interactor and predicted 12 potential SIMs in RAD54L2 (*23*–*25*).

RAD54L2 has been described as a transcriptional coregulator and is a member of the RAD54 subfamily of sucrose nonfermentable (SNF2)–type chromatin remodeling factors (*26*, *27*). Previous biochemical characterization unveiled it as a DNA-dependent adenosine triphosphatase (ATPase) (*28*). To dissect which properties of RAD54L2 are relevant in relation to TOP2-mediated DNA damage, we generated an ATPase catalytic site mutant (K311A), as previously characterized (*26*, *29*), and a ΔSIM mutant, point-mutated at the predicted SIMs identified in previous work (Fig. 2C and fig. S2C) (*23*). We monitored the etoposide sensitivity of *RAD54L2*^KO^ cells complemented with mCherry alone, mCherry-RAD54L2^WT^, or mCherry-RAD54L2^ΔSIM^ by clonogenic survival assays (Fig. 2D). Notably, while expression of RAD54L2^WT^ complemented the etoposide hypersensitivity of *RAD54L2*^KO^ cells—and actually made them more resistant than CTRL cells—ATPase catalytic site or ΔSIM RAD54L2 mutant proteins did not complement the etoposide sensitivity (Fig. 2D). These data thus implied that the ATPase activity and SIMs of RAD54L2 are crucial for its role(s) in responding to TOP2-mediated DNA damage.

To explore these protein variants biochemically and to validate the IP-MS results, we coimmunoprecipitated mCherry from extracts of RPE-1 cells stably expressing mCherry alone [empty vector (EV)], mCherry-RAD54L2^WT^, or mCherry-RAD54L2^ΔSIM^ mutant and grown in the presence or absence of etoposide (Fig. 2E). We observed that RAD54L2^WT^ interacted with TOP2α and TOP2β, with binding to high molecular weight modified forms of these proteins being dramatically enhanced upon etoposide treatment. By contrast, while RAD54L2^ΔSIM^ still interacted with lesser/unmodified forms of TOP2α and TOP2β, binding to etoposide-induced modified forms of TOP2α and TOP2β was no longer evident. Furthermore, we noted that, in line with our IP-MS data, binding of RAD54L2^WT^ to ZNF451 was stimulated upon etoposide treatment, although this was not the case for the RAD54L2^ΔSIM^ mutant. Knowing that TOP2α and TOP2β are expressed at different levels in different phases of the cell cycle, we tested whether RAD54L2 interaction with TOP2 proteins is cell cycle regulated by performing IPs from extracts of G_1_- or S-phase synchronized cells. As expected, TOP2α expression was much lower in G_1_ compared to S phase, meaning that we could only readily detect its interaction with RAD54L2 in S phase (fig. S2D). However, TOP2β expression was essentially equal throughout the cell cycle, allowing us to show that RAD54L2 interacted similarly with TOP2β in both G_1_ and S phase cells (fig. S2D). Overall, these results indicated that RAD54L2 likely affects responses to TOP2ccs throughout the cell cycle.

### RAD54L2 operates in a pathway dependent on ZNF451 and independent of TDP2

**Fig. 3.**
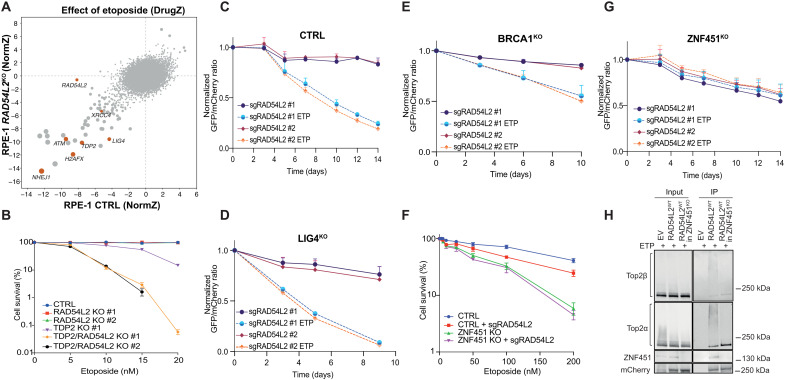
RAD54L2 operates through a mechanism dependent on ZNF451 and independent of TDP2. (**A**) Biplot showing gene enrichment scores (NormZ) upon etoposide treatment in *TP53*^KO^ (*x* axis) and *TP53/RAD54L2*^KO^ (*y* axis) RPE-1 cells. Orange dots relate to well-characterized DDR factors, most discussed in the text. (**B**) Clonogenic survival assays of CTRL, *RAD54L2*^KO^, *TDP2*^KO^, and *TDP2/RAD54L2*^KO^ RPE-1 cells upon treatment with etoposide. *n* = 3 independent experiments. Bars represent means ± SEM. Competitive growth assays of RPE-1 CTRL (**C**), *LIG41*^KO^ (**D**), *BRCA1*^KO^ (**E**), and *ZNF451*^KO^ (**G**) cells transduced with virus expressing the indicated sgRNAs in the presence of various etoposide concentrations; *n* = 3 independent experiments. Bars represent means ± SEM. (**F**) Clonogenic survival assays of CTRL or *ZNF451*^KO^ transduced with a CTRL sgRNA or with an sgRNA targeting *RAD54L2*. *n* = 3 independent experiments. Bars represent means ± SEM. (**H**) Extracts of etoposide-treated CTRL or *ZNF451*^KO^ RPE-1 cells stably expressing mCherry [empty vector (EV)] or mCherry-RAD54L2^WT^ were subjected to mCherry IP followed by Western blotting for the indicated proteins. This experiment was repeated three times with similar results.

Covalent TOP2-DNA adducts are known to be removed either directly, through the concerted actions of specialized nucleases, or in a TDP2-dependent manner. To explore where RAD54L2 acts in relation to known TOP2-DNA adduct repair processes and to unveil potential novel genetic interactions, we performed genome-scale CRISPR-Cas9 gene inactivation screens in RPE-1 *TP53*^KO^ (CTRL) and *RAD54L2/TP53*^KO^ cells (fig. S3A) in the presence or absence of etoposide. Ensuing bioinformatics analyses indicated that, as expected, *RAD54L2* was a screen dropout hit in the CTRL background but not in the *RAD54L2*^KO^ background. Our analyses revealed that *RAD54L2* was not epistatic with any other factors scoring in the screens and known to be involved in promoting repair of TOP2-DNA adducts, including TDP2, ATM, and NHEJ components such as NHEJ1, x-ray repair cross complementing 4 (XRCC4), and ligase 4 (LIG4) (Fig. 3A; note that in the CRISPR library used, sgRNAs targeting ZNF451 were likely noneffective since they were not depleted upon etoposide treatment despite this factor’s well-established roles in the response to etoposide treatment). To validate the screen results, we generated human RPE-1 and HAP1 *TDP2*^KO^ and *TDP2/RAD54L2*^KO^ cells (fig. S3, B and C) and tested them for etoposide hypersensitivity via clonogenic survival assays and 3-(4,5-dimethylthiazol-2-yl)-2,5-diphenyltetrazolium bromide (MTT)–based cell viability assays (Fig. 3B and fig. S3D). In accord with the screening data, *RAD54L2*^KO^ and *TDP2*^KO^ cells showed mild and moderate etoposide hypersensitivity, respectively, while the double *TDP2/RAD54L2*^KO^ displayed a notable hypersensitivity phenotype indicating synergistic effects of TDP2 and RAD54L2. Similarly to the *RAD54L2*^KO^ cells, *TDP2*^KO^ and *TDP2/RAD54L2*^KO^ cells were not hypersensitive to ionizing radiation in either RPE-1 or HAP1 backgrounds (fig. S3, E and F).

To validate the genetic relationships between RAD54L2 and the HR and NHEJ pathways observed in our CRISPR screens, we performed competitive cell growth assays in *LIG4*^*K*O^ cells (fig. S3G) or breast cancer 1 (*BRCA1*)*^KO^* cells (*30*), as models of NHEJ and HR deficiency, respectively. Thus, we transduced CTRL, *LIG4*^*K*O^, or *BRCA1^KO^* cells with lentiviral vectors containing either of two different sgRNAs targeting *RAD54L2* and monitored the relative growth of the gene-edited populations over time in the presence of selected doses of etoposide. As expected from our CRISPR screen data, inactivation of *RAD54L2* sensitized CTRL, *LIG4*^*K*O^, and *BRCA1^KO^* cells to etoposide (Fig. 3, C to E, and fig. S3, H, J, and K). Collectively, these experiments suggested that RAD54L2 operates in a hitherto unrecognized pathway for TOP2ccs.

Having found that RAD54L2 likely interacts with sumoylated TOP2 via its SIMs, and considering that we observed the SUMO E3 ligase ZNF451 as a strong RAD54L2 interactor after etoposide treatment, we tested whether ZNF451 and RAD54L2 act in the same pathway. To monitor whether RAD54L2 loss enhanced the etoposide sensitivity of CTRL or *ZNF451*^KO^ cells (fig. S3I), we performed clonogenic survival assays in CTRL or *ZNF451*^KO^ cells transduced with sgRNAs targeting *RAD54L2.* We observed that CTRL cells transduced with sgRNAs targeting *RAD54L2* became hypersensitive to etoposide (Fig. 3F). However, *ZNF451*^KO^ cells transduced with sgRNAs targeting *RAD54L2* were not further sensitized to etoposide, implying an epistatic relationship between ZNF451 and RAD54L2. In addition, competitive cell growth assays in these same cell lines confirmed that sgRNA targeting *RAD54L2* hypersensitized CTRL but not *ZNF451*^KO^ cells to etoposide (Fig. 3G and fig. S3L). Collectively, these data supported a model in which RAD54L2 acts in processes controlled by the SUMO E3 ligase ZNF451 in response to etoposide.

In line with the above findings, we observed that interactions between RAD54L2 and etoposide-induced posttranslationally modified forms of TOP2α and TOP2β were essentially abolished when cells were preincubated with the sumoylation inhibitor ML-792 (fig. S3M). Furthermore, by performing IPs of RAD54L2 from extracts of CTRL or *ZNF451*^KO^ cells, we observed that RAD54L2 was not able to effectively interact with etoposide-induced post-translationally modified TOP2α and TOP2β in the absence of ZNF451, while it still interacted with unmodified TOP2 (Fig. 3H). Collectively, these data are in accord with there being key functional connections between ZNF451 and RAD54L2 in response to etoposide treatment.

### RAD54L2 counters TOP2ccs formation and prevents DSB generation upon etoposide treatment

**Fig. 4.**
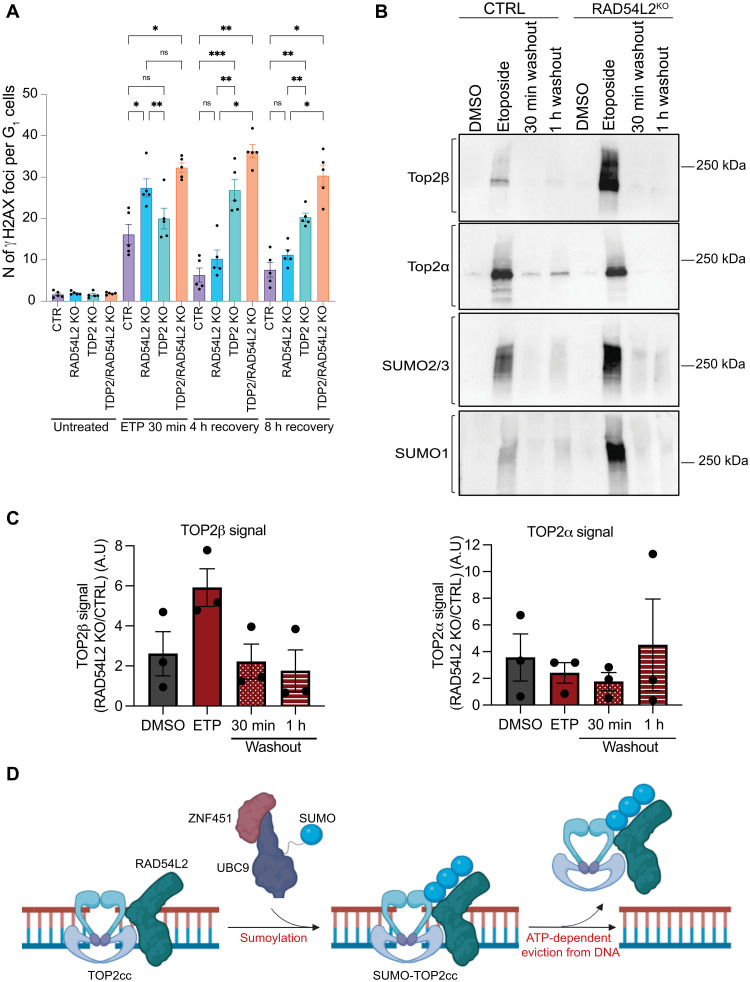
RAD54L2 counters TOP2ccs by promoting their resolution. (**A**) Quantification of γH2AX foci in G_1_ cells (cyclin A negative). Cells were treated with 30 μM etoposide for 30 min, washed, and left to recover for 4 or 8 hours. *n* > 3 independent experiments. Bars represent means ± SEM. One-way analysis of variance (ANOVA) was used for statistical testing. (**B**) DUST assays in CTRL or *RAD54L2*^KO^ HAP1 cells collected after 1 hour of treatment with 20 μM etoposide or 30 min or 1 hour after etoposide washout. This experiment was carried out three times with similar results. (**C**) Quantifications of TOP2α or TOP2β signal from the DUST assays. Bars represent means ± SEM. (**D**) Proposed mechanism for RAD54L2 in TOP2cc resolution. UBC9, SUMO conjugating enzyme 2 ligase. Figure generated with BioRender. ns, not significant.

To investigate cellular readouts of such a mechanism, we tested levels of chromosomal DSB formation and resolution in CTRL, *RAD54L2*^KO^, *TDP2*^KO^, and *TDP2/RAD54L2*^KO^ cells by analyzing formation of γH2AX foci (markers of DSBs) after etoposide treatment (Fig. 4A). Notably, *RAD54L2*^KO^ cells started with significantly higher numbers of γH2AX foci than CTRL and *TDP2*^KO^ cells. Upon removal of etoposide, however, *RAD54L2*^KO^ cells resolved damage at a much faster rate than *TDP2*^KO^ cells, implying that RAD54L2 functions specifically on TOP2cc resolution and not DSB repair. The *TDP2/RAD54L2^KO^* cells displayed higher levels of γH2AX throughout the experiment, reinforcing our conclusion that RAD54L2 and TDP2 act synergistically and in different pathways.

We next used the detection of ubiquitylated and sumoylated TOP-DNA-protein cross-links (DPCs) (DUST) assays to monitor levels and SUMO posttranslational modifications of trapped TOP2α and TOP2β (*8*). In this assay, only proteins covalently bound to DNA copurify with nucleic acids and are readily detected by Western blotting using antibodies targeting SUMO1, SUMO2/3, TOP2α, or TOP2β. We observed that in the presence of etoposide, levels of SUMO1- or SUMO2/3-conjugated DPCs and trapped TOP2β were higher in *RAD54L2*^KO^ cells compared to CTRL cells (Fig. 4, B and C). Overall, these data thus supported a role for RAD54L2 in affecting TOP2cc levels.

## DISCUSSION

Collectively, our findings support a model in which RAD54L2 interacts with TOP2α and TOP2β, recognizes SUMO-modified TOP2ccs through its SIMs, and uses its ATPase activity to help resolve TOP2ccs and prevent DSB exposure. Furthermore, we have demonstrated that RAD54L2 acts with ZNF451 but in processes distinct from those mediated by TDP2, whose role in removing TOP2ccs is also promoted by ZNF451 activity. Our work thus identifies a key function for RAD54L2 and adds yet another layer of complexity to cellular mechanisms that safeguard the genome from TOP2-mediated DNA damage (Fig. 4D), which occur not only in response to etoposide but also under normal physiological conditions. Since *TDP2* gene mutations have been linked to hereditary neurological disease in individuals with seizures, ataxia, and intellectual disability (*31*, *32*), we speculate that RAD54L2 may protect against neurological disorders. In addition, in line with our finding that overexpression of RAD54L2 in WT cells generates etoposide resistance (Fig. 2D), high expression of RAD54L2 was recently associated with inferior event-free and overall survival of acute myeloid leukaemia (AML) patients in the AML02 cohort (*33*–*35*). As RAD54L2-mediated TOP2cc resolution could represent a mechanism for intrinsic or acquired tumor resistance to chemotherapies including TOP2 poisons, it represents a potential biomarker and a candidate target for anticancer drug discovery.

## MATERIALS AND METHODS

### Cell culture and cell line generation

RPE-1 WT cells were originally obtained from J. Pines. RPE-1 *TP53*^KO^ cells were generated as described previously (*36*). HAP1-Cas9 and RPE-Cas9 *RAD54L2* knockout cells were generated by transduction of a RAD54L2 dual-guide RNA plasmid (guide A: ATTGGACAGCTGGTAGTAGG, guide B: GTACCCTCACCTCATCACCA). For genome-wide screens, *RAD54L2* knockout cells were generated by transient transfection of a synthetic guide RNA (gRNA) targeting *RAD54L2* (CTCTTCTTGCTGTGCTGCTT) in RPE-1 *TP53*^KO^ cells. *TDP2* knockout cells were generated by transient transfection of a synthetic gRNA targeting *TDP2* (GTAGAAATATCACATCT). *LIG4* knockout cells were generated by transient transfection of a synthetic gRNA targeting *LIG4* (GGAAGAATTAGTCTCATTGC) in RPE-1 *TP53*^KO^ cells. *ERCC6L2*^KO^ cells were purchased from Horizon (ID: HZGHC006416c004). RPE-1 cells were cultured at 37°C and 5% CO_2_ in Dulbecco’s modified Eagle’s medium:nutrient mixture Ham’s F-12 (DMEM/F-12, Sigma-Aldrich) supplemented with 17 ml of 7.5% NaHCO_3_ per 500 ml (Sigma-Aldrich), 10% (v/v) fetal bovine serum (FBS; BioSera), penicillin (100 U/ml), streptomycin (100 μg/ml) (Sigma-Aldrich), and 2 mM l-glutamine. Blasticidin (10 μg/ml; Sigma-Aldrich) was used to select for Cas9-expressing cells. Cells were additionally cultured with puromycin (1.5 μg/ml) during selection of the transductants in the CRISPR-Cas9 screens. HAP1 cells were cultured at 37°C and 5% CO_2_ in Iscove’s modified Dulbecco’s medium (Thermo Fisher Scientific) supplemented with 10% (v/v) FBS (BioSera), penicillin (100 U/ml), streptomycin (100 μg/ml) (Sigma-Aldrich), and 2 mM l-glutamine. LentiX 293T and U2OS cells were cultured at 37°C and 5% CO_2_ in DMEM (Sigma-Aldrich). RPE-1 cells were synchronized in G_1_ phase by treating with 5 μM palbociclib (S1116-SEL, Stratech Scientific Ltd.) for 20 hours. S-phase synchronization was obtained by treating with 2 mM thymidine (T1895, SLS Limited) for 20 hours and then releasing in fresh medium for 2 hours. When indicated, 10 μM ML-792 (HY-108702, MedChemExpress) was added for 30 min before etoposide treatment.

### CRISPR-Cas9 screens

For DDR-focused CRISPR-Cas9 screens, biological duplicates of RPE-1 WT and *TP53*^KO^ Cas9-expressing cells were transduced at a multiplicity of infection (MOI) of 0.3- and 1000-fold coverage of the custom dual-gRNA DDR library as described previously (*15*). Afterward, transductants were selected with puromycin for 12 days before treatment with dimethyl sulfoxide (DMSO) or etoposide [drug concentration reducing cell viability by 50% (IC_50_)] for 7 days with three additional days without any drug. Library preparation and next-generation sequencing of the samples were performed as described previously (*15*). Guide enrichment analysis was performed using DrugZ to compare DMSO-treated to etoposide-treated samples (*37*).

For the genome-wide CRISPR-Cas9 screens, RPE-1 *TP53*^KO^ or RPE-1 *TP53/RAD54L2*^KO^ Cas9-expressing cells were transduced at an MOI of 0.2- and 500-fold coverage of the Gattinara library (*38*). Afterward, transductants were selected with puromycin for 8 days and either left untreated or treated with etoposide (IC_30_) for 15 days. Genomic DNA from cell pellets was isolated using the QIAamp Blood Maxi Kit (Qiagen), and genome-integrated sgRNA sequences were amplified by polymerase chain reaction (PCR) using the Q5 Mastermix (New England Biolabs Ultra II) and i7 multiplexing barcoded primers. The final gel-purified products were sequenced on Illumina NovaSeq 6000 systems. Guide enrichment analysis was performed using DrugZ to compare DMSO-treated to etoposide-treated samples (*37*).

### MTT viability and clonogenic survival assays

For MTT cell viability assay, 1000 HAP1 cells were plated in 96-well plates in triplicate and treated with the indicated drugs 24 hours later. Four days later, the medium was removed, and cells were incubated with MTT (0.5 mg/ml). Four hours later, 10% SDS was added, and absorbance was read the day after at 595 nm. For clonogenic assays, 200 RPE-1 cells were plated in six-well plates, treated with drugs 24 hours later, and stained and counted 10 to 12 days later, when visible colonies were formed. Cells were treated with etoposide (VP-16, Cayman Chemicals), carboplatin (C2538, Sigma-Aldrich), camptothecin (C9911, Sigma-Aldrich), 5-aza-2′-deoxycytidine (Merck, A3656), formaldehyde (Sigma-Aldrich, F1635), olaparib/AZD2281 (Stratech Scientific, A4154), ICRF-193 (GR332, BIOMOL International), or hydroxyurea (H8627, Sigma-Aldrich).

### Competitive cell growth assay

CTRL or *ZNF451*^KO^ human RPE-1 cells were transduced (MOI >1) with mCherry-sgLacZ or green fluorescent protein (GFP)–sgRAD54L2 (#1 or #2). Transduced cells were selected with puromycin 2 days after transduction and cultured for 2 more days until non-transduced cells were dead. In six-well plates, 50,000 cells expressing mCherry-sgLacZ were mixed with 50,000 cells expressing GFP-sgRAD54L2 (#1 or #2) and plated with DMSO or etoposide. Cell mixtures were collected at the indicated days and subcultured (to prevent confluency) into fresh medium containing DMSO or etoposide. Cell mixtures were analyzed by fluorescence-activated cell sorting (Beckman Coulter, Cytoflex S) for cells expressing GFP or mCherry. The ratio of GFP^+^/mCherry^+^ cells was analyzed with FlowJo v9. To calculate the efficiency of indel formation after sgRNA transduction, DNA was extracted from cells 4 days after transduction and regions near the cut site were PCR-amplified, sequenced, and analyzed by TIDE (Netherlands Cancer Institute, http://shinyapps.datacurators.nl/tide/).

### Plasmid, siRNA transfection, and viral transduction

The mCherry plasmid was obtained from VectorBuilder (VB200726-1045daz). Cloning of RAD54L2 cDNA into the mCherry vector was done by Gibson assembly (New England Biolabs) following PCR of RAD54L2 using the forward primer 5′-AGGATGACGATGACAAGAGCTCAGACGAATCTGCCTCAGG and reverse primer 5′-TCGAGGTCGACACGCGTGTTTTTCCCAGTGACCTCTATCAC and vector digestion with Hpa I/Afe I (New England Biolabs) restriction enzymes. RAD54L2 K311A mutant was generated by mutagenesis using primers 5′-GAGATCACTTGCAAAGTTGCCCCCAGACCCATGCTGTG and 5′-CACAGCATGGGTCTGGGGGCAACTTTGCAAGTGATCTC. RAD54L2 ΔSIM mutant (mutant details in table S2) was generated by gene synthesis (GeneWiz). The synthesized open reading frame was then cloned into the mCherry vector by Gibson assembly using the same conditions as above with the reverse primer 5′-CGAGGTCGACACGCGTGTTTTTCCCAGTGGCCTCTGCCGC. To generate stable cells, virus was first produced in LentiX 293T cells by cotransfecting the packaging constructs psPAX2 (Addgene, #12260) and pMD2.G (Addgene, #12259) with the plasmid of interest using TransIT-LT1 (Mirus Bio) according to the manufacturer’s protocol. Viral particles were then incubated with cells in the presence of polybrene (10 μg/ml) (Merck), followed by positive selection with geneticin (Gibco).

### Immunofluorescence and microscopy imaging

For imaging purposes, cells were plated in 24-well imaging plates, treated with etoposide (30 μM, 30 min), washed, and released for the indicated period of time. After treatments, cells were fixed in 4% paraformaldehyde for 10 min, permeabilized in phosphate-buffered saline (PBS)–0.2% Tween for 10 min, and blocked in PBS–5% bovine serum albumin (BSA) for at least 30 min. After 1 hour of incubation at room temperature with primary antibodies (γH2AX, Cell Signaling Technology, 2577 and cyclin A, BD Biosciences, 611268), cells were washed in PBS–0.2% Tween three times and incubated with secondary antibodies for 45 min. After three more washes in PBS–0.2% Tween, nuclei were stained with 4′,6-diamidino-2-phenylindole for 10 min. Images were acquired and analyzed using the Opera Phoenix microscope.

### Immunoblotting

Cells were lysed in Laemmli buffer [2% SDS, 10% glycerol, and 60 mM tris-HCl (pH 6.8)], incubated for 5 min at 95°C, and resolved by SDS–polyacrylamide gel electrophoresis (SDS-PAGE). After transferring onto a nitrocellulose membrane, the membranes were blocked with 5% milk or BSA in TBS-T buffer (tris-buffered saline with Tween 20, 0.1%) and incubated with primary antibodies diluted in 5% milk or BSA in TBS-T. The membranes were washed in TBS-T and probed with secondary antibodies. After secondary antibody incubation, membranes were washed in TBS-T, incubated with enhanced chemiluminescence (ECL) mixture for 5 min in the dark, and developed using films or a ChemiDoc imaging system (Bio-Rad). Antibodies used were as follows: RAD54L2 (Abcam, ab86063), TOP2α (Bethyl Laboratories, A300-054A), TOP2β (BD Biosciences, 611493), mCherry (Abcam, ab167453), tubulin (Sigma-Aldrich, T9026), TDP2 (Bethyl, A302-737A), ZNF451 (Sigma-Aldrich, SAB2108741), LIG4 (ab193353, Abcam), and glyceraldehyde-3-phosphate dehydrogenase (GAPDH) (ab8245, Abcam).

### Immunoprecipitation

For IP, around 2 million RPE-1 cells were plated in 15-cm dishes (2 per condition). Two days later, the cells were treated with 100 μM etoposide for 1 hour. After treatment, the cells were washed twice with cold PBS and lysed in 1.5 ml of IP buffer [20 mM tris-HCl (pH 7.5), 150 mM NaCl, 2 mM MgCl_2_, 10% glycerol, 0.5% NP-40, 20 mM *N*-ethylmaleimide (NEM), and EDTA-free protease and phosphatase inhibitors] and 15 μl of benzonase (Millipore) for 45 min. Lysates were centrifuged at 15,000 rpm for 10 min, and supernatants were incubated with 20 μl of RFP-Trap magnetic beads (ChromoTek) for 2 hours at 4°C. The samples were washed 4× with IP buffer and finally eluted in 40 μl of lithium dodecyl sulfate (LDS) buffer 2× + 1 mM dithiotheitol (DTT).

### MS-based proteomics

Bound proteins were eluted in NuPAGE LDS Sample Buffer (Life Technologies) supplemented with 1 mM DTT and boiled at 75°C for 15 min. The eluates were alkylated with 5.5 mM chloroacetamide for 30 min in the dark before being loaded onto 4 to 12% gradient SDS-PAGE gels. Proteins were stained with the Life Technologies Colloidal Blue Staining Kit and digested in-gel using trypsin. Peptides were extracted from the gel and desalted on reversed-phase C18 StageTips. Peptide fractions were analyzed on a quadrupole Orbitrap mass spectrometer (Q Exactive or Q Exactive Plus, Thermo Fisher Scientific) equipped with an ultrahigh-performance liquid chromatography system (EASY-nLC 1000, Thermo Fisher Scientific) as described (*39*, *40*). Peptide samples were loaded onto C18 reversed-phase columns (15 cm length, 75 μm inner diameter, 1.9 μm bead size) and eluted with a linear gradient from 8 to 40% acetonitrile containing 0.1% formic acid over 2 hours. The mass spectrometer was operated in data-dependent mode, automatically switching between MS and MS^2^ acquisition. Survey full-scan MS spectra [mass/charge ratio (*m/z*) 300 to 1700] were acquired in the Orbitrap. The 10 most intense ions were sequentially isolated and fragmented by higher-energy C-trap dissociation (HCD) (*41*). An ion selection threshold of 5000 was used. Peptides with unassigned charge states, as well as with charge state less than +2, were excluded from fragmentation. Fragment spectra were acquired in the Orbitrap mass analyzer.

### Analysis of MS data

Raw data files were analyzed using MaxQuant (development version 1.6.14.0) (*42*). Parent ion and MS2 spectra were searched against a database, and peptide lists were searched against the human UniProt FASTA database released in February 2021 using the Andromeda search engine (*43*). Spectra were searched with a mass tolerance of 6 parts per million (ppm) in MS mode, 20 ppm in HCD MS^2^ mode, strict trypsin specificity, and allowing up to three miscleavages. Cysteine carbamidomethylation was searched as a fixed modification, whereas protein N-terminal acetylation and methionine oxidation were searched as variable modifications. The dataset was filtered on the basis of posterior error probability to arrive at a false discovery rate (FDR) of below 1% estimated using a target-decoy approach (*44*). The “match between run algorithm” in the MaxQuant quantification was enabled (*45*). The MaxLFQ protein groups data calculated by MaxQuant were further analyzed with the Perseus (v1.6.13.0) of the MaxQuant computational platform (*46*). Identified peptides were filtered for potential contaminants and reverse reads localization probability (>75%). Missing values were replaced by random numbers drawn from a normal distribution with a width of 0.3 and down shift of 1.8. Protein groups with no proteotypic and less than two peptide identifications were excluded. Only protein groups identified in three of five replicates in at least one treatment group were included. Label-free protein quantitation (LFQ) was performed with a minimum ratio of 2 (*47*). LFQ intensities normalized by the MaxLFQ algorithm were log_2_-transformed and calculated for the average log_2_(FC) (fold change). *P* values were calculated by Student’s *t* test (*48*). For RAD54L2 interactors, protein groups with fold change 2 and *P* value ≤0.05 were considered significant interactors. Volcano plots were made in RStudio (v1.3.1093) with customized scripts. For pathway enrichment analysis, significant interactors (FDR < 0.05) from the untreated IP-MS experiments (RAD54L2-UT/RFP-UT) were used for a statistical overrepresentation test with the whole human genome as reference using PANTHER GOslim biological process annotations (http://pantherdb.org/). Significant interactors linked to DNA replication in the untreated samples were identified as significantly enriched using the Reactome (v80) pathway browser (https://reactome.org). Full results and enrichment analyses are available (table S3 and S4).

### DUST assay

The DUST assay (*8*) is an extension of the RADAR assay developed by Maizels and colleagues for detecting proteins covalently bound to DNA (*49*, *50*). The DUST assay is an extension of the assay that also allows detection of posttranslational modifications on proteins covalently bound to DNA by using antibodies directed against the modifications. The RADAR purification scheme eliminates proteins that are not covalently bound to nucleic acids. For the experiments described here, cells were grown in 60 mM dishes to around 50 to 70% confluence. After specific treatment conditions as described, the medium was removed by aspiration and cells were resuspended in 600 μl of DNAzol (Invitrogen), which also contained 1× protease inhibitor cocktail (Sigma-Aldrich), 1 mM DTT, and 20 mM NEM (Sigma-Aldrich). After 10 min of incubation at 4°C with gentle rocking, 300 μl of ice-cold 100% ethanol was added to each plate and the plate was agitated until a white precipitate formed. The lysate was transferred to a microcentrifuge tube and centrifuged at maximum speed for 15 min at 4°C. The supernatant was removed, and the pellet was washed with 75% ethanol and spun for 2 min. All the supernatant was removed, and the pellet was air-dried briefly. The pellets were resuspended in 200 μl of tris-ethylenediaminetetraacetic acid (EDTA) (TE) buffer overnight at 4°C. The following day, the samples were sonicated for 10 s two times using the Branson 450 Sonifier with a small probe and set at 30% power. The DNAs were quantitated using the Qubit dsDNA BR Assay. Then, 5 μg of DNA was digested by adding CaCl_2_ to a final concentration of 5 mM and 1 μl of micrococcal nuclease (Thermo Fisher Scientific) and incubating the samples for 30 min at 37°C digest DNA; RNA is also digested by micrococcal nuclease, eliminating the need for a ribonuclease step. The reactions were stopped by adding Laemmli buffer and boiled for 5 min. The samples were loaded on 4 different 4 to 15% TGX Mini-protean precast gels (Bio-Rad) and each indicated protein was probed on a separate blot. After wet transfer to polyvinylidene difluoride, the membranes were blocked and incubated with the indicated antibodies [TOP2α (Bethyl Laboratories, A300-054A), TOP2β (BD Biosciences, 611493), SUMO2/3 (ab3742, Abcam), SUMO1 (Abcam ab11672)] overnight at 4°C. The following day, the membranes were washed and incubated with secondary antibodies [GE Healthcare, NAV931V, ECL–anti-mouse horseradish peroxidase (HRP)–linked antibody; GE Healthcare, NAV9340V, ECL–anti-rabbit HRP-linked antibody] and visualized using Femto or Atto ECL reagents (Thermo Fisher Scientific).

### Statistics and reproducibility

Graphs and statistical tests were made using Prism v9 (GraphPad Software). One-way analysis of variance (ANOVA) was used to analyze the data in Fig. 4A.

### Figures and schematics

Schematics were made using BioRender (https://biorender.com/), and figures were made with Adobe Illustrator.
