# Supplementary material for: RAD54L2 counters TOP2-DNA adducts to promote genome stability
Source: Sci Adv. 2023 Dec 6;9(49):eadl2108. doi: 10.1126/sciadv.adl2108 (PMC10699776; doi:10.1126/sciadv.adl2108)
Supplement: Supplementary file 1 — Figs. S1 to S3 Tables S1 and S2 Legends for tables S3 and S4 [file sciadv.adl2108_sm.pdf]

Science Advances
AAAS

Supplementary Materials for

RAD54L2 counters TOP2-DNA adducts to promote genome stability

Giuseppina D'Alessandro *et al.*

Corresponding author: Giuseppina D'Alessandro, giuseppina.dalessandro@cruk.cam.ac.uk;
Guido Zagnoli-Vieira, guido.zagnolivieira@astrazeneca.com; Stephen P. Jackson, steve.jackson@cruk.cam.ac.uk

Sci. Adv. **9**, eadl2108 (2023)
DOI: 10.1126/sciadv.adl2108

The PDF file includes:

Figs. S1 to S3
Tables S1 and S2
Legends for tables S3 and S4

Other Supplementary Material for this manuscript includes the following:

Tables S3 and S4

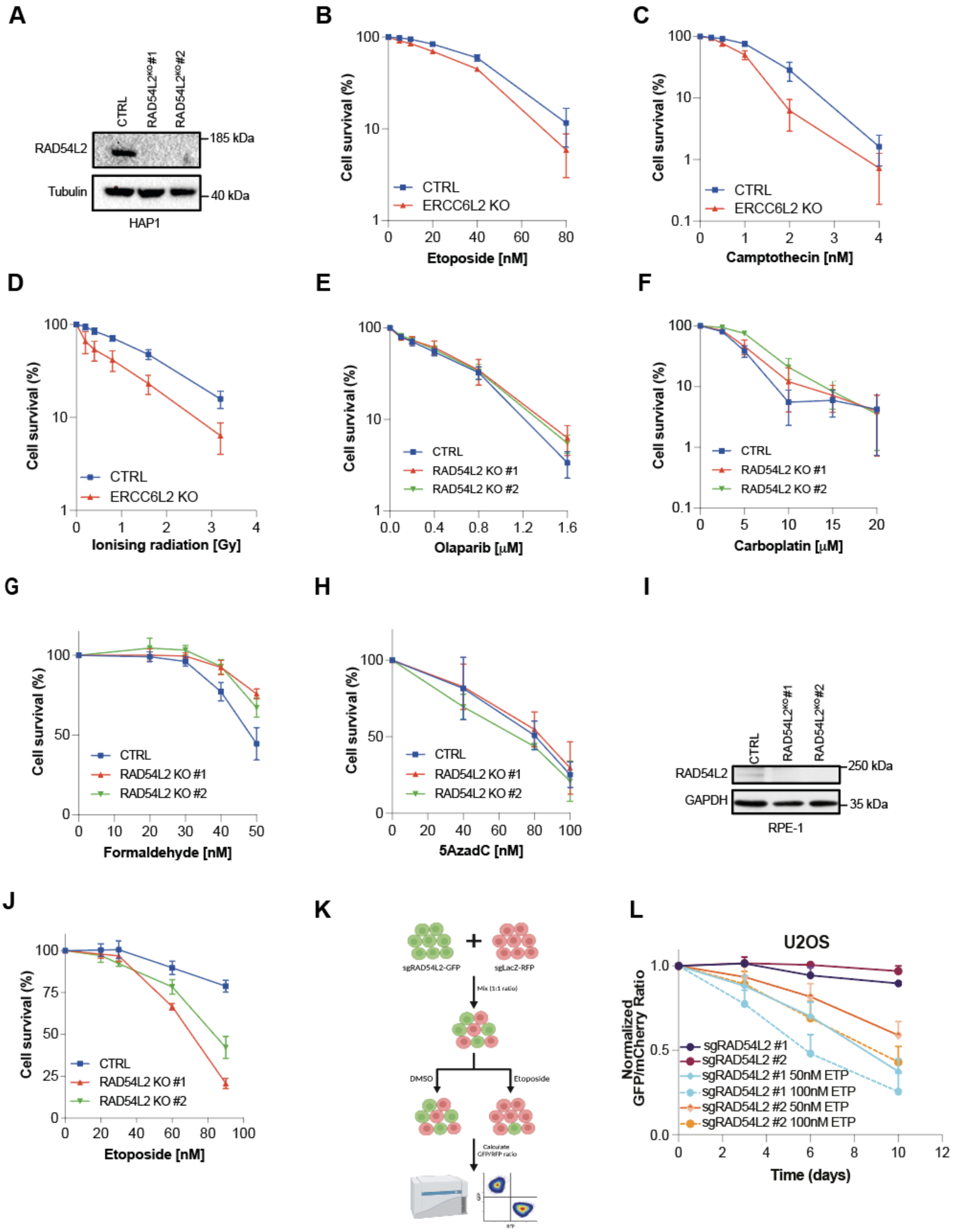

Fig. S1. RAD54L2 loss confers hypersensitivity to etoposide but not to other DNA damaging agents tested. (**A**) Western blot validation of *RAD54L2*^{KO} in HAP1 cells. (**B-D**) MTT viability assay of *ERCC6L2*^{KO} HAP1 cells upon treatment with etoposide (**B**), camptothecin (**C**) or ionizing radiation (**D**). n=3 independent experiments. Bars represent means ± SEM. (**E-H**) Viability assay of two *RAD54L2*^{KO} HAP1 clones upon treatment with olaparib (**E**), carboplatin (**F**), formaldehyde (**G**), or 5-aza-2'-deoxycytidine (**H**); n=3 independent experiments. Bars represent means ± SEM. (**I**) Western blot validation of *RAD54L2*^{KO} in RPE-1 cells. (**J**) Clonogenic survival assays of two *RAD54L2*^{KO} RPE-1 clones upon etoposide treatment. n=3 independent experiments. Bars represent means ± SEM. (**K**) Schematic of the competitive cell growth assay. Figure generated with BioRender. (**L**) Competitive growth assays of U2OS cells stably expressing Cas9 transduced with virus expressing the indicated sgRNAs in the presence of etoposide; n=3 independent experiments. Bars represent means ± SEM.

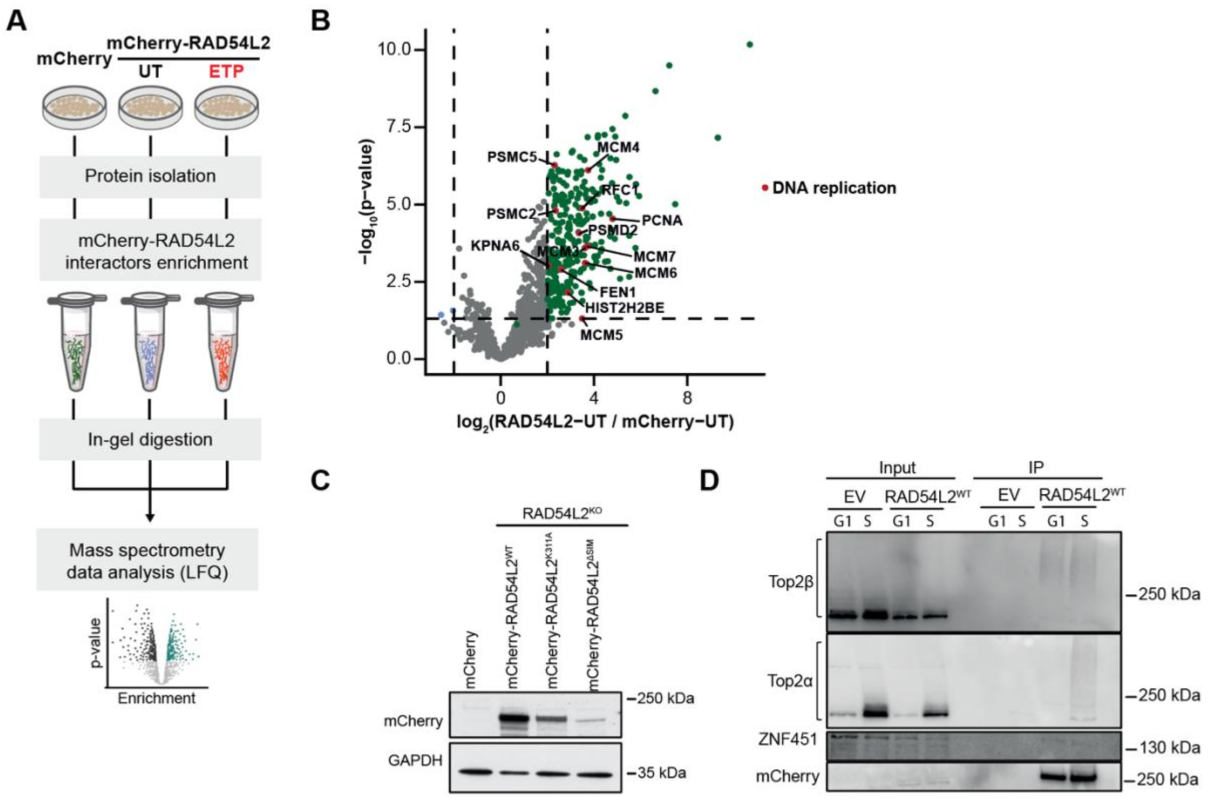

Fig. S2. RAD54L2 interactors identified by IP-MS. (**A**) Schematic of IP-MS experiments in human RPE-1 cells expressing mCherry or mCherry-RAD54L2^{WT}. Figure generated with BioRender. (**B**) Volcano plot showing RAD54L2 interactors identified by IP-MS in untreated (UT) cells. Green dots marked by dotted lines indicating p-values ≤0.05 and log_2 fold changes ≥2 are significant interactors. Significant interactors linked to DNA replication (red dots) were identified using the Reactome(v80) pathway browser. (**C**) Western blot showing RAD54L2 expression in RPE-1 *RAD54L2*^{KO} cells complemented with RAD54L2^{WT}, RAD54L2^{K311A} , or RAD54L2$^{\Delta SIM}$ constructs. (**D**) Co-immunoprecipitation (IP) of mCherry in etoposide treated *RAD54L2*^{KO} cells stably expressing mCherry (empty vector, EV) or mCherry-RAD54L2^{WT} synchronised in G1- or S-phase. This experiment was repeated three times with similar results.

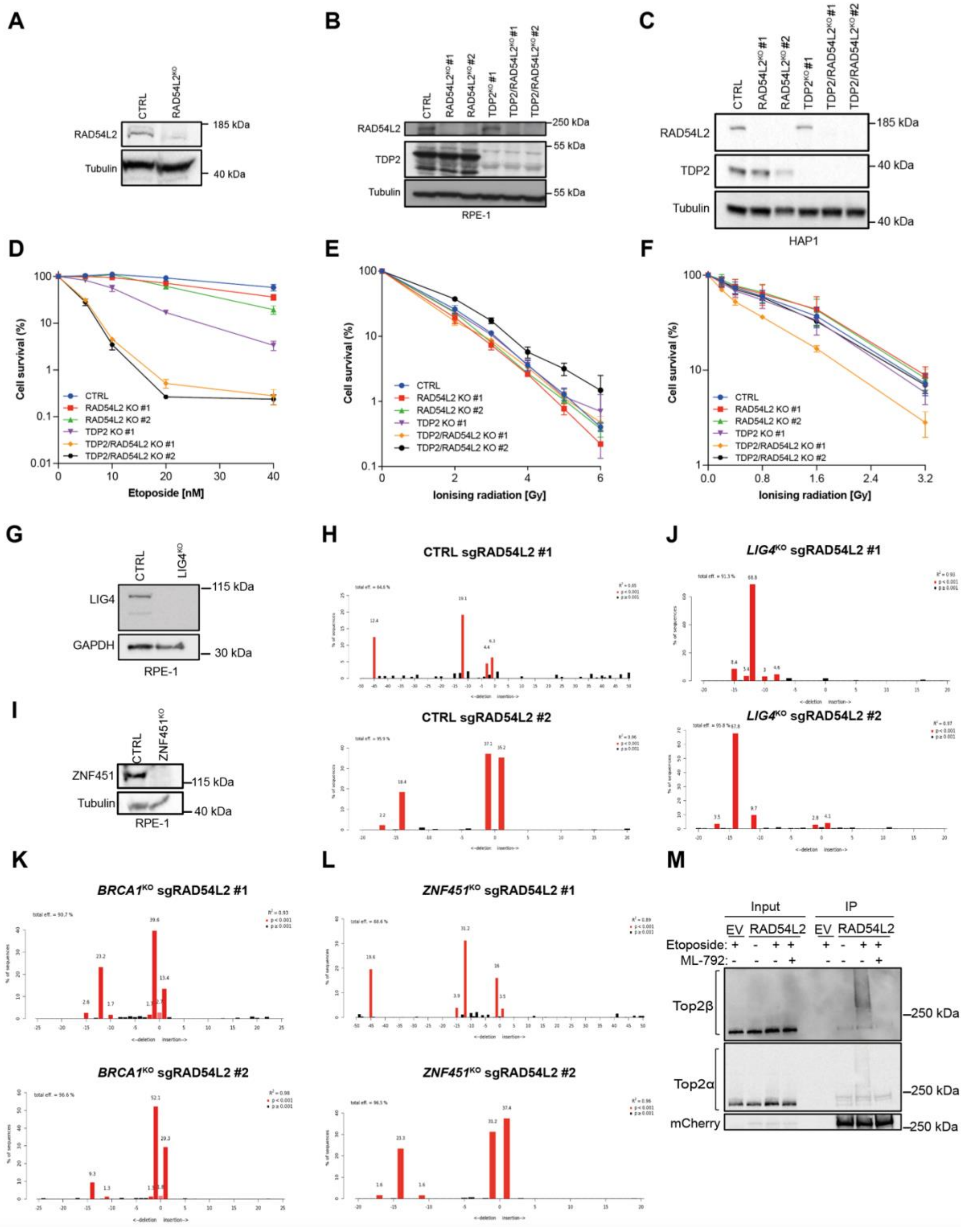

Fig. S3. RAD54L2 acts with ZNF451 but in a different pathway than TDP2. (**A**) Western blot validation of *RAD54L2*^{KO} in RPE-1 *TP53*^{KO} cells. (**B-C**) Western blot validation of *RAD54L2*^{KO} and *TDP2*^{KO} in RPE-1 (**B**) and HAP1 (**C**) cells. (**D**) MTT viability assays of *RAD54L2*^{KO}, *TDP2*^{KO}, and *TDP2/RAD54L2*^{KO}HAP1 cells upon etoposide treatment; n=3 independent experiments. Bars represent means ± SEM. (**E-F**) Clonogenic (**E**) or MTT viability (**F**) assays of *RAD54L2*^{KO}, *TDP2*^{KO}, and *TDP2/RAD54L2*^{KO} RPE-1 (**E**) or HAP1 (**F**) cells upon treatment with ionizing radiation; n=3 independent experiments. Bars represent means ± SEM. (**G-I**) Western blot validation of *LIG4*^{KO} (**G**) or *ZNF451*^{KO} (**I**) in RPE-1 *TP53*^{KO} cells. (**H-L**) TIDE analysis of *RAD54L2* Cas9-editing in CTRL (**H**), *LIG4*^{KO} (**J**), *BRCA1*^{KO} (**K**), or *ZNF451*^{KO} (**L**) cells 4 days post-transduction. (**M**) Extracts of etoposide-treated RPE-1 cells stably expressing mCherry (empty vector, EV) or mCherry-RAD54L2^{WT} were subjected to mCherry immunoprecipitation followed by Western blotting for the indicated proteins. This experiment was repeated two times with similar results.

Primers for mutagenesis	
Fw_K311A	GAGATCACTTGCAAAGTTGCCCCCAGACCCATGCTGTG
Rev_K311A	CACAGCATGGGTCTGGGGGCAACTTTGCAAGTGATCTC
Oligos for competition assay	
Fw1 _sgRAD54L2	CACCGGTTGACAAGGACCCGCCCAA
Rev1_sgRAD54L2	AAACTTGGGCGGGTCCTTGTCAACC
Fw2 _sgRAD54L2	CACCGTCTGATTGGTGCCAACCGAG
Rev2_sgRAD54L2	AAACCTCGGTTGGCACCAATCAGAC
TIDE PCR primers for competition assay	
Fw1 _sgRAD54L2	TTCACTCCCTGGATTTTTGG
Rev1_sgRAD54L2	TACGGAGTAGGGCAAACCTG
Fw2 _sgRAD54L2	GCCTTCTGCCATCGTAATGC
Rev2_sgRAD54L2	ATGTGCCTCTCCGAAGTCTG

Table S1.
Oligonucleotides

Mutation	AA location	Protein domain
**K > A**	311	ATPase site (activity)
**VICLD > AACLD**	182	1 SIM
**VIELS > AAELS**	201	2 SIM
**LHIVD > LHAAD**	212	3 SIM
**IVPVN > AAPAN**	334	4 SIM
**IIDLD > AADLD**	432	5 SIM
**VVICD > AAACD**	459	6 SIM
**VILVR > AALAR**	578	7 SIM
**IVIPG > AAAPG**	1077	8 SIM
**IHIIR > AHAAR**	1098	9 SIM
**IISEL > AASEL**	1175	10 SIM
**VLDLR > ALDLR**	1248	11 SIM
**VIEVT > AAEAT**	1461	12 SIM

Table S2.
List of RAD54L2 mutations

Table S3.
RAD54L2 interactome results following IP-MS

Table S4.
GOslim enrichment analysis of biological processes of RAD54L2 interactome
